# The Host Cell Sulfonation Pathway Contributes to Retroviral Infection at a Step Coincident with Provirus Establishment

**DOI:** 10.1371/journal.ppat.1000207

**Published:** 2008-11-14

**Authors:** James W. Bruce, Paul Ahlquist, John A. T. Young

**Affiliations:** 1 Institute for Molecular Virology, University of Wisconsin, Madison, Wisconsin, United States of America; 2 Howard Hughes Medical Institute University of Wisconsin, Madison, Wisconsin, United States of America; 3 Infectious Disease Laboratory, The Salk Institute for Biological Studies, La Jolla, California, United States of America; Aaron Diamond AIDS Research Center, United States of America

## Abstract

The early steps of retrovirus replication leading up to provirus establishment are highly dependent on cellular processes and represent a time when the virus is particularly vulnerable to antivirals and host defense mechanisms. However, the roles played by cellular factors are only partially understood. To identify cellular processes that participate in these critical steps, we employed a high volume screening of insertionally mutagenized somatic cells using a murine leukemia virus (MLV) vector. This approach identified a role for 3′-phosphoadenosine 5′-phosphosulfate synthase 1 (PAPSS1), one of two enzymes that synthesize PAPS, the high energy sulfate donor used in all sulfonation reactions catalyzed by cellular sulfotransferases. The role of the cellular sulfonation pathway was confirmed using chemical inhibitors of PAPS synthases and cellular sulfotransferases. The requirement for sulfonation was mapped to a stage during or shortly after MLV provirus establishment and influenced subsequent gene expression from the viral long terminal repeat (LTR) promoter. Infection of cells by an HIV vector was also shown to be highly dependent on the cellular sulfonation pathway. These studies have uncovered a heretofore unknown regulatory step of retroviral replication, have defined a new biological function for sulfonation in nuclear gene expression, and provide a potentially valuable new target for HIV/AIDS therapy.

## Author Summary

A genetic screen was used to identify host cell functions important for the replication of retroviruses, including human immunodeficiency viruses. These studies have uncovered a heretofore unexpected role for the cellular sulfonation pathway in an intracellular step of retroviral replication. Through the addition of sulfate groups, this pathway is responsible for modifying and regulating different types of cellular factors including proteins, lipids, carbohydrates and hormones. The role of this pathway was further confirmed by using specific chemical inhibitors. The sulfonation requirement was mapped to a step during viral DNA integration into the host genome that has a subsequent effect upon the level of expression of viral genes. These studies have uncovered a new regulatory mechanism of retroviral replication and suggest that components of the host cell sulfonation pathway might represent attractive targets for antiviral development.

## Introduction

The *Retroviridae* are a large viral family that includes the human pathogens Human Immunodeficiency Viruses 1 and 2 (HIV-1 and HIV-2), the causative agents of acquired immune deficiency syndrome (AIDS). Due to their small coding capacity and requirement for integration into the host cell genome, retroviruses are heavily dependent upon host cell machinery for efficient replication. The retroviral lifecycle can be divided into two distinct phases. The early stage consist of virus binding to a cellular receptor, fusion of viral and cellular membranes leading to delivery of the viral core into the cytoplasm, reverse transcription of the positive strand RNA genome to generate a dsDNA product, translocation of viral nucleoprotein complexes to the nucleus, and provirus establishment through integration of the viral DNA into the host cell genome. The late stage consists of transcription of the viral genome by host RNA pol II, RNA processing and export to the cytoplasm, translation of viral proteins, viral assembly, egress and maturation.

While progress has been made on the identification of many of the cellular proteins involved in the late stage of the retroviral lifecycle, particularly in transcription, RNA processing and egress, less is known about the contribution of cellular factors to the early stage of the retroviral lifecycle. In particular, the contribution of cellular factors to steps subsequent to virus:cell membrane fusion and that lead to proviral DNA establishment are only partially understood [1]. A number of cellular factors that facilitate early steps in infection have been identified, although in some cases the roles of these factors are controversial. These factors include the actin cytoskelton and microtubule network [2]–[7], LAP-2α, barrier-to-autointegration factor (BAF), and emerin [8]–[15], SUMOylation factors [16], importins [17]–[19], tRNAs [20] and LEDGF [21]–[28]. Although a recent genome-wide siRNA screen uncovered a number of cellular genes that contribute to various stages of HIV infection, it was notable that only a few additional factors were described that are associated with either viral DNA synthesis or integration [17]. It therefore seems likely that other, as yet unidentified, cellular factors participate in early retroviral replication.

To identify other cellular factors that are involved, we have employed a somatic cell mutagenesis-based approach. This study led to the identification of the 3′-phosphoadenosine 5′-phosphosulfate synthase 1 (PAPSS1) gene as playing an important role in retroviral replication. PAPSS1 and PAPSS2 are homologous enzymes that synthesize 3′-phosphoadenosine 5′-phosphosulfate (PAPS), the high energy sulfate donor used in all known sulfonation reactions catalyzed by cellular sulfotransferases [29]. Golgi sulfotransferases catalyze the sulfonation of lipids, of carbohydrates, and of tyrosines in proteins [29]–[33]. Cytoplasmic sulfotransferases lead to the sulfonation of a wide variety of peptides, hormones and xenobiotics [29],[34]. The data described in this report reveal a novel role for the cellular sulfonation pathway in retroviral replication during provirus establishment, one that modulates the subsequent transcriptional competency of the provirus.

## Materials and Methods

### Plasmids and viral vectors

A schematic of the proviral forms of the MLV constructs used in this paper is provided in Figure S1. The viral genome plasmids pMMP-nls-LacZ, pCMMP-eGFP and pCMMP-IRES-GFP, pCMMP-CD4-eGFP, pHIV-TVA800-hcRED, pRET and the ASLV-A genome plasmid RCASBP(A)-AP have been previously described [35]–[37]. The MLV vectors pLEGFP (Clontech, Palo Alto, CA) and pQCLIN (Clontech, Palo Alto, CA) as well as the HIV-1 self inactivating (SIN) pLenti6/V5-GW/lacZ (Invitrogen, Carlsbad, CA) were obtained commercially. The HIV-1 vector pNL4-3.Luc.R-E- [38] was obtained from the NIH AIDS Research and Reference Reagent Program, Division of AIDS, NIAID, NIH (deposited by Dr. Nathaniel Landau).

To construct the MLV vector pCMMP-CD4 (expressing human CD4 from the viral LTR), the previously described pCMMP-CD4-eGFP vector [39] was digested with PmlI and HpaI to remove the IRES-eGFP cassette and then the plasmid was re-ligated. The MLV vector pCMMP-HcRED (encoding the red fluorescent protein HcRED from the viral LTR) was generated by removing the multiple cloning site and IRES from pCMMP-IRES-GFP by AgeI/HpaI digestion and inserting an AgeI/StuI fragment containing the HcRED coding sequence from pHcRED1 (Clontech, Palo Alto, CA). The MLV vector pCMMP-SEAP-IRES-GFP (encoding SEAP and GFP) was generated by inserting the SEAP gene from pSEAP-control (Clontech, Palo Alto, CA) upstream of the IRES in pCMMP-IRES-GFP. HIV-1 vectors for stable expression of PAPS synthases or control cDNAs were generated by PCR amplification of coding sequence (PAPSS1: IMAGE#3869484, PAPSS2: IMAGE#2988345, control cDNA ZNF639:IMAGE#4794621) from commercially available cDNAs (Open Biosystems) and cloning into MluI/EcoRV digested pLenti6/V5-GW/lacZ.

### Cell culture and virus production

Chinese hamster ovary cells (CHO-K1, ATCC CCL-61) were cultured in F-12 media supplemented with 10% bovine calf serum (BCS) (Invitrogen, Carlsbad, CA). Human embryonic Kidney 293T cells (ATCC CRL-11268) were cultured in DMEM supplemented with 10% fetal calf serum (FCS) (Hyclone, Logan, UT). Chicken DF-1 cells (ATCC CRL-12203) were cultured in DMEM supplemented with 10% FCS. Jurkat cells (ATCC TIB-152) were cultured in RPMI-1640 supplemented with 10% FCS. CHO cells expressing the receptor for ASLV (TVA-800) were generated as previously described [39] by infection with HIV-1-TVA800-hcRED[VSV-G] at an approximate moi of 0.5 hcRED transducing units for 2 hours. Cells infected with this virus express: TVA800; HcRED; and the blasticidin S deaminase (BSD) gene. Infected cells were selected for two weeks in the presence of 3 µg/ml blasticidin (Invitrogen, Carlsbad, CA). Cells expressing either PAPSS1, PAPSS2 or the control cDNA, ZNF639, were generated by infecting CHO-K1 and IM2 cells with VSV-G pseudotyped HIV-1 vectors encoding the appropriate ORF (see above) at an approximate moi of 0.5 blasticidin transducing units for 2 hours. Infected cells were selected for two weeks in 3 µg/ml blasticidin. MLV VSV-G and EnvA pseudotyped viruses were generated by calcium phosphate transfection of 293T cells as previously described [35],[39],[40]. VSV-G pseudotyped HIV-1 vector was produced by a similar proceedure except the genome plasmid used was pNL4-3.Luc.R-E-. The VSV-G pseudotyped self-inactivating HIV-1 vector was made using the Virapower kit (Invitrogen, Carlsbad, CA) following manufacturers instructions. DF-1 cells were transfected using the calcium phosphate method with the subgroup A-specific ASLV-A vector, RCASBP (A)-AP, encoding alkaline phosphatase [37]. Media from transfected cells was collected 2 days post transfection to 7 days post transfection and filtered through a 0.45 µm bottle top filter. Virus was stored at 4°C through the collection period, combined and then frozen at −80°C for long-term storage. Virus for use in Quantitative PCR amplification studies was treated with DNaseI (Roche Applied Science, Indianapolis, IN) to remove contaminating plasmid DNA from the virus preps. DNaseI was added as a powder to a final concentration of 1 µg/ml when the virus containing supernatants were collected. The supernatants were incubated 1 hr at room temperature before filtration. The titer of VSV-G and envA vector stocks were determined by assaying for transduction of a marker gene following infection of either WT CHO-K1 cells or WT CHO-K1 cells that had been engineered to express TVA800 [39]. For viruses that lack a cell-based reporter gene assay, immunoblot analysis of viral capsid protein (CA) levels (α-p24 for HIV or α-p36 for MLV) in the extracellular supernatants of producer cells was used to equalize the amounts of input virus as compared to those associated with viral vectors that contain reporter genes (Lenti6/V5-GW/lacZ[VSV-G] for HIV and MMP-nls-LacZ[VSV-G] for MLV).

### Retroviral Insertional mutagenesis and isolation of an MLV-resistant clone

CHO-K1 cells (1×10^8^) were mutagenized by infection with VSV pseudotyped pRET at an approximate MOI of 0.01 GFP transducing units. Cells were selected in 900 µg/ml G418 for two weeks. A pool of 2×10^7^ insertionally mutagenized CHO-K1 cells were challenged with CMMP-CD4 [VSV-G] at an approximate m.o.i of 1 CD4 transducing units for two hours at 37°C 37 in the presence of 4 µg/ml polybrene. Unbound viruses were then removed and fresh medium was added. At 48 hours post infection (hpi) the cells were removed from the plate with phosphate buffered saline (PBS) containing 5 mM EDTA. Cells were pelleted (200×g, 5 min) and resuspended in 500 µl PBS containing 2 mM EDTA and 2% bovine serum albumin (BSA) (Sigma-Aldrich, Inc., St. Louis, MO). The cells were incubated with anti-human CD4 iron-conjugated antibody (Miltenyi Biotec Inc., Auburn, CA) at 20 µg/10^7^ cells for 15 minutes at 4°C. Large cell (LC) columns (Miltenyi Biotec Inc., Auburn, CA) were applied to a magnetic field and washed with 2 ml PBS containing 2 mM EDTA and 2% BSA. Cells were filtered through a 30 µm mesh (Miltenyi Biotec Inc., Auburn, CA) and applied to the LC column. Cells were washed twice with 2 ml PBS containing 2 mM EDTA and 2% BSA. Column flow through and washes were collected and the cells were pelleted, resuspended in medium and replated. Cells were allowed to recover for at least 16 hours before the next viral challenge. When necessary, the cells were expanded between each round of virus challenge to a minimum of 5×10^5^ cells per sort. The challenge and selections were repeated five times. The population was challenged a final time with CMMP-HcRED[VSV-G] and the HcRed negative cells were single cell cloned after high speed FACS (University of Wisconsin Comprehensive Cancer Center).

Single cell clones from the sorted insertional mutant pools were grown for 14 days post sorting, trypsinized and then plated onto duplicate assay plates. The assay plates were incubated for 2 hours with pMMP-nls-LacZ [VSV-G] at an approximate m.o.i. of 1 LacZ transducing unit (LTU) in the presence of 4 µg/ml polybrene. Unbound virus was then removed and fresh medium was added. At 48 hpi, one plate was assayed for β-galactosidase activity using the Galacto-Star chemiluminescent kit (Applied Biosystems, Foster City, CA) according to the manufacturers instructions and the other plate was assayed for cell number and cell viability using CellTiter-Glo reagent (Promega, Madison, WI) following the manufacturers instructions to control for variations in cell number among the clones.

### Chemiluminescent assay of viral infection

Quantitative chemiluminescent infection assays were performed as previously described [39], briefly, 8 wells of a 96 well plate were seeded at 1×10^4^ cells/well for each cell line tested. The cells were incubated for 2 hours with an approximate m.o.i of 1 transducing unit (based on marker gene expression for β-galactosidase and alkaline phosphatase, or CA equivalents for luciferase, as described above), in the presence of 4 µg/ml polybrene. Unbound virions were removed and fresh medium was added. At 48 hpi, four wells were assayed for β-galactosidase activity using the Galacto-star Kit (Applied Biosystems, Foster City, CA), for alkaline phosphatase activity using the Phospha-Light Kit (Applied Biosystems, Foster City, CA) or for luciferase activity using the Britelite (PerkinElmer, Boston, MA) according to the manufacturer's instructions. The other four wells were assayed for cell number and cell viability using CellTiter-Glo reagent (Promega, Madison, WI) as described above. The results obtained were normalized for relative cell number.

To determine the absolute fold-resistance to viral infection, X-Gal staining was performed on cells that were infected with serial dilutions of viruses. For these experiments, cells were seeded at 1×10^4^ cells/well in triplicate rows for each cell line tested. The cells were then infected for 2 hours with ten-fold serial dilutions of MMP-nls-LacZ [VSV-G] in the presence of 4 µg/ml polybrene as described before and the cells were subsequently stained with X-gal as previously described [41]. The blue cells contained in wells that had between 20 and 200 β-galactosidase positive cells were counted to give an accurate measure of the viral titer.

### PAPS assays

PAPS assays were performed as previously described [42]. Briefly, cells were lysed by three freeze thaw cycles in PAPS lysis buffer [20 mM Tris pH 8, 20% sucrose, 1 mM EDTA, 1 mM DTT] in the presence of 1× protease inhibitor cocktail (RPI, Mt. Prospect, IL). Cell lysate (1 µl) was mixed with 5 mM ATP and 10 μCi ^[35]^S labeled sulfate in reaction buffer [50 mM Tris pH 8, 25 mM MgCl_2_, 0.9 M EDTA, 13.5 mM DTT) and incubated for 30 minutes at room temperature. Thin layer chromatography (TLC) was used to separate PAPS, APS and SO_4_ on PEI cellulose TLC plate (EMD Chemicals, Gibbstown, NJ) in 0.9 M LiCl. TLC plates were dried, exposed to phosphoimager plates, and quantified using the Imagequant software volume method. Mobility positions were confirmed with commercial PAPS preparations (PerkinElmer, Waltham MA, Cat# NEG010100UC). Each sample was normalized for µg of total protein in the lysate determined by Bradford assay using the Quick Start Bradford Dye reagent (Bio-rad, Hercules, CA).

### Real time quantitative PCR

To measure the amounts of reverse transcription intermediates in infected cells, cells were seeded in triplicate wells at 5×10^5^/well in a 6 well plate and then infected at 4°C on a rocking platform at an m.o.i. of 1 GFP transducing unit (GTU) for 2 hours with an MLV vector (pLEGFP; Clontech, Palo Alto, CA) pseudotyped with VSV-G that was treated with DNaseI as described above. Virus derived from pLEGFP was used for these assays because the 3′ viral LTR varied enough from pCMMP so realtime PCR primers could be designed that specifically recognized the pLEGFP derived test virus but not the pCMMP derived screen virus. DNA was harvested from infected cells 24 hpi (hpi) using the DNeasy Kit (Qiagen, Valencia, CA). For the nuclear fractionation studies nuclei were harvested from infected cells 24 hpi using the Nuclei EZ Prep Kit (Sigma-Aldrich, Inc., St. Louis, MO) following the manufacturers instructions and DNA was isolated from nuclei as described above. To measure integrated proviral DNA copy number, cells were seeded and infected as described above and then passaged for 18 days. DNA was then harvested from 1×10^6^ cells as described above. DNA concentration was calculated by measuring the A260 on a SPECTRAmax Plus 96 well UV spectrophotometer (Molecular Devices, Sunnyvale, CA). Quantitative, real time PCR (QPCR) analysis was performed on an ABI 9600 (Applied Biosystems, Foster City, CA) using the standard cycling conditions of 50°C 10 min, 40 cycles of 95°C 30 s, 60°C 2 minutes. DNA (10 µl/25 µl reaction) was amplified in TaqMan Universal PCR Mastermix (Applied Biosystems, Foster City, CA) with 1 µM each primer and 0.1 µM 5′, 6-FAM, 3′TAMRA labeled probe. Each primer probe set was tested on each cell line in a minimum of 3 independent experiments. The number of molecules in each reaction was determined by comparison to standard curves generated from amplification of plasmid DNA containing the target sequence. The primers used are specific for the U3-U5 region of the LEGFP vector and are shown along with the viral LTR feature and the bp position recognized in pLEGFP are: OJWB39 (5′-CAGTTCGCTTCTCGCTTCTGTTC-3′) [U3, bp 523–535], OJWB47 (5′-GTCGTGGGTAGTCAATCACTCAG-3′) [R and U5, bp 697–719] and OJWB38 (5′-6-FAM- ATCCGAATCGTGGTCTCGCTGTTC-TAMRA-3′) [R, bp 657–680].

### RNase Protection Assays

Templates for RNA probes to MLV were generated by PCR amplification using 1 µg total DNA from CHO-K1 cells infected with CMMP-GFP[VSV-G] along with the oligonucleotide primers OJWB7 (5′-GAACAGATGGTCCCCAGATGC-3′) and OJWB8 (5′-CGGTGGAACCTCCAAATGAA-3′). ExTaq polymerase (Takara, Madison WI) was used with cycling conditions of [95°C 5 min, 30 cycles of 95°C 30 S, 50°C 30 S 72°C 1 min]. This resulting LTR fragment was cloned into pGem T-easy (Promega, Madison, WI) and spanned 192 bp upstream of the transcription start (+1, the start of R) to 139 bp downstream of +1, which results in a 140 bp protected fragment in the RNase protection assays. The template for RNA probes to hamster actin RNA were generated by reverse transcription of the hamster β-actin cDNA cloned by reverse transcription PCR amplification of 1 µg total RNA isolated from CHO-K1 cells with OJWB313 (5′- TCACCCACACTGTGCCCATCTATGA-3′) and OJWB314 (5′-CAACGGAACCGCTCATTGCCAATGG-3′) and MasterAmp tTh polymerase (Epicenter, Madison WI) using cycling conditions of [60°C 5 min, 30 cycles of 95°C 30 S, 50°C 30 S 72°C 1 min]. The resulting PCR amplified product was cloned into pGem T-easy (Promega, Madison, WI) and generates a 294 bp protected fragment in RNase protection assays. Anti-sense RNA probes were generated by digesting the plasmids with *Spe*I and performing performing in vitro transcription reaction using the Riboscribe Kit (Epicenter, Madison WI) with T7 polymerase and 50 μCi α−^[32]^P-UTP.

To measure the amounts of transcription from integrated proviruses in infected cells, cells were seeded in triplicate wells at 5×10^5^/well in a 6 well plate and then infected at 4°C on a rocking platform at an m.o.i. of 1 GTU for 2 hours with an MLV vector (pCMMP-GFP) pseudotyped with VSV-G. RNA was isolated form cells 24 hpi using the RNeasy kit (Qiagen, Valencia, CA) following manufacturers instructions. RNase protection assays were performed by mixing 2 µg (viral transcripts) or 0.5 µg (β-actin) of total RNA with 5×10^4^ cpm probe, hybridizations and digestions were done using the RPA III kit (Ambion, Ausin TX). Protected fragments were separated on a 6% PAGE-Urea gel, dried and exposed to a phosphoimager plate. Phosphorimage units were measured using the Imagequant software volume method.

## Results

### Isolation of the IM2 cell line resistant to infection by a MLV vector

Chinese hamster ovary (CHO-K1) cells were used for insertional mutagenesis by a retroviral vector since these cells are functionally hypodiploid at numerous loci [43] and therefore insertion of the viral vector into a single allele of a given cellular gene can be sufficient to produce a genetically-null phenotype. The insertional mutagenesis was performed with the murine leukemia virus (MLV)-based vector pRET, which encodes green fluorescent protein (GFP), as well as a neomycin phosphotransferase (NPT) mRNA that contains an instability element downstream of a canonical splice donor site [44]. Integration of pRET upstream of a cellular exon gives rise to a NPT mRNA transcript in which the instability element is removed by mRNA splicing, thereby conferring G418 resistance on the mutagenized cells (Figure S1A).

**Figure 1:**
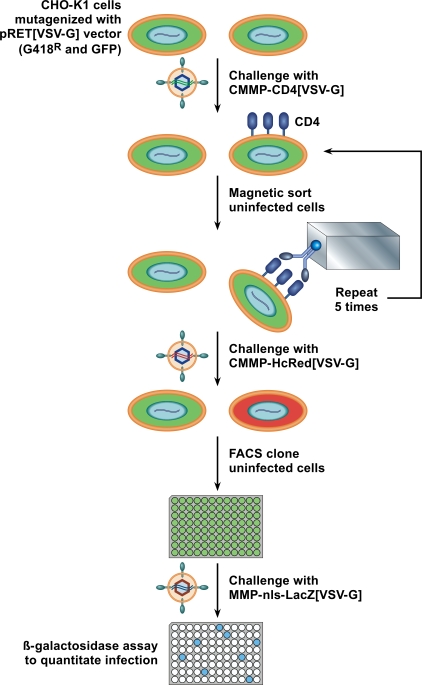
Scheme used to isolate pRET-mutagenized CHO-K1 cell lines that are resistant to subsequent retroviral infection. CHO-K1 cells were mutagenized by infection with a VSV-G pseudotyped pRET vector at a moi of 0.01 G418^R^ transducing units and selected in G418 for 2 weeks. Pools of mutagenized cells (1×10^7^) were challenged with a VSV-G pseudotyped MLV vector that encodes CD4. Infected cells were depleted from the population by magnetic sorting with an iron conjugated anti-CD4 antibody. After five rounds of challenge and sorting, the enriched pools were infected with a VSV-G pseudotyped MLV vector that encodes the red fluorescent protein HcRed. The non-fluorescent cells were single cell cloned by FACS, expanded and seeded into duplicate assay plates. The assay plates were infected with another VSV-G pseudotyped MLV vector that encodes β-galactosidase. One plate was then assayed with a chemiluminescent assay for β-galactosidase. For control purposes, the other plate was assayed with a luciferase based chemiluminescent assay to measure viable cell number.

**Figure 2:**
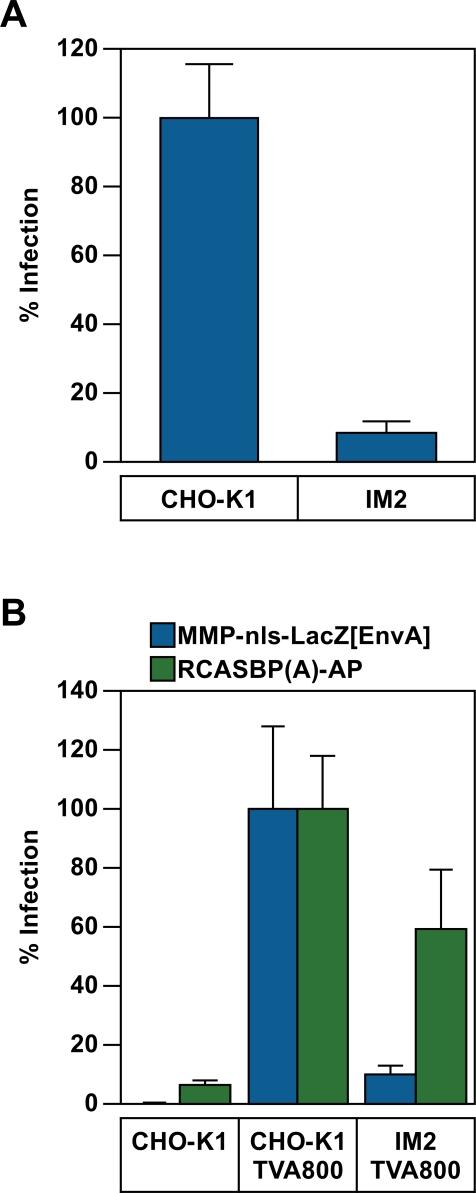
Resistance of the IM2 cell line maps to the MLV core. (A) CHO-K1and IM2 cells were challenged with serial dilutions of the VSV-G pseudotyped MLV vector (MMP-nls-lacZ[VSV-G]), encoding β-galactosidase. The cells were then stained 48 hpi with X-gal, the number of blue cells were counted, and the data reported as the percentage of LacZ transducing units (LTU) obtained from WT CHO-K1 infections (5×10^5^ LTU). The data shown are the average of three experiments each performed with triplicate samples. Error bars indicate the standard deviation of the data. (B) CHO-K1 cells and IM2 cells, engineered to express TVA800, or wild-type CHO-K1 cells, were challenged with either pMMp-nls-LacZ[envA], an EnvA pseudotyped MLV vector encoding β-galactosidase, or with RCASBP(A)-AP, an ALSV-A vector encoding heat stable alkaline phosphatase. Infection was monitored using chemiluminescent assays to detect reporter enzyme activities along with a chemiluminescent assay to measure relative viable cell numbers. The ratios of [enzyme activities∶relative viable cell number] were calculated for each sample and compared with values from CHO-K1 TVA 800 cells (defined as 100% infection). The data shown are the average mean values obtained in an experiment performed with quadruplicate samples and are representative of three independent experiments. Error bars indicate the standard deviation of the data.

Approximately 1×10^6^ colonies of G418-resistant cells were generated by challenging CHO-K1 (1×10^8^) cells with VSV-G pseudotyped pRET at an moi of 0.01 (note: at this moi only a small fraction of these cells are “infected”) to ensure only one integration event per cell. Mutagenized cells were selected in medium containing 900 µg/ml G418 for two weeks, after which the population was expanded and pooled. In order to identify cells in the population that were resistant to retroviral infection, a pool of 2×10^7^ insertionally mutagenized cells were subjected to five rounds of challenge with a second, replication-defective, VSV-G pseudotyped MLV vector which contains a human CD4 gene that is expressed from the viral promoter (Fig. 1). Infected cells that expressed human CD4 on their surface were removed from the population at each round by magnetic cell sorting (MACS) using an iron-conjugated CD4-specific antibody (Fig. 1). Each round of infection and sorting resulted in an approximate 3-fold enrichment of CD4-negative cells relative to the preceding round, with a total enrichment of 47-fold. The resultant cell population, which exhibited an overall 2.5-fold resistance to MLV infection, was then challenged a final time with another VSV-G pseudotyped MLV vector encoding the far-red fluorescent protein HcRed. A total of 264 single cell clones of HcRed-negative cells were then isolated by FACS (Fig. 1) and tested for their susceptibility to infection by a VSV-G pseudotyped MLV vector encoding β-galactosidase. One cell line, designated IM2, that was judged to be one of the most resistant (approximately 12-fold) to challenge by that viral vector, based upon viral reporter gene expression (Fig. 2A), is characterized in detail in this report.

To determine if the defect associated with the IM2 cell line is specific for the MLV vector, wild-type CHO-K1 cells and mutant IM2 cells were engineered to express TVA800, the cellular receptor for an avian retrovirus, subgroup A avian sarcoma and leukosis viruses (ASLV-A) [45],[46]. The TVA800 expressing cells were then challenged with either the MLV vector encoding β-galactosidase vector pseudotyped with the ASLV-A envelope protein (EnvA) or instead with an ASLV-A vector that encodes heat-stable alkaline phosphatase [37]. Viral reporter gene expression following infection of IM2-TVA800 cells by the EnvA-pseudotyped MLV vector was 9.7-fold reduced as compared with CHO-K1-TVA800 cells (Fig. 2B). This effect mirrored that seen with VSV-G pseudotyped MLV vectors (e.g. Fig. 2A). Thus, the defect seen with IM2 cells is independent of the nature of the viral glycoprotein used to pseudotype the MLV vector. By contrast, the level of viral reporter gene expression following infection by the ASLV-A vector was comparable between IM2-TVA800 and CHO-K1 cells (Fig. 2B). Since both vectors utilized EnvA to mediate entry, these observations indicate that the defect associated with the IM2 cell line is specific for protein or RNA components of the MLV core.

### The PAPSS1 gene is disrupted in IM2 cells

**Figure 3:**
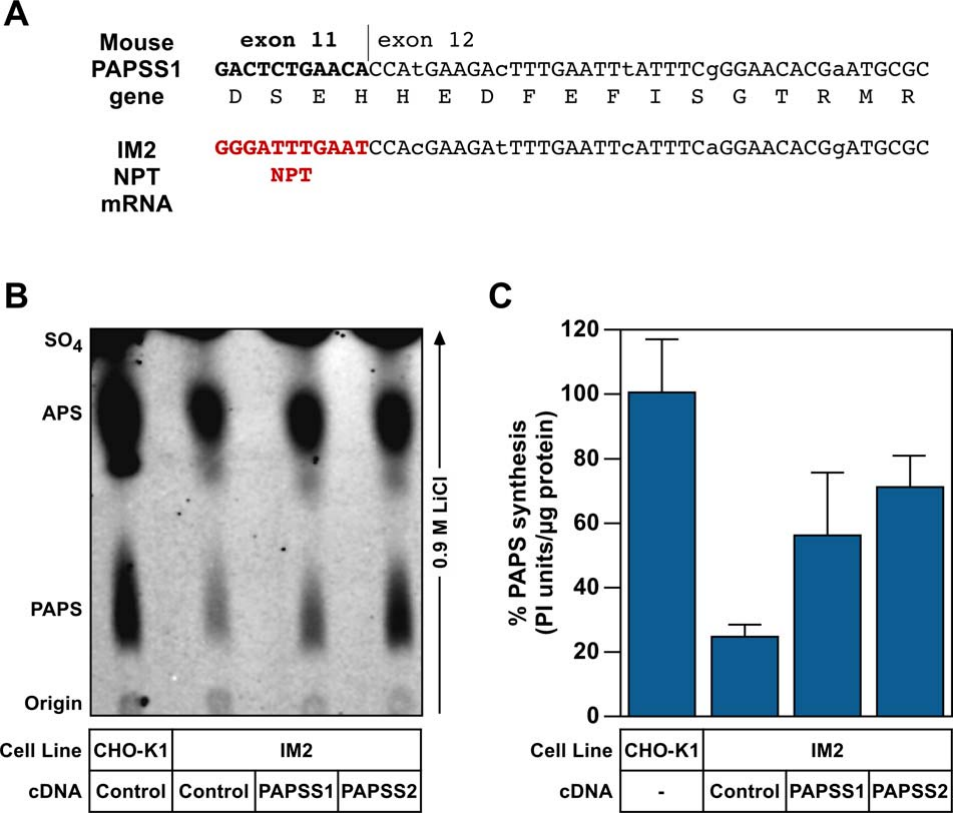
PAPS synthase 1 gene is disrupted in IM2 cells. (A) The pRET mutagenic vector is integrated upstream of exon 12 of the PAPSS1 gene. The nucleotide sequence of the fusion junction that is formed via mRNA splicing involving the splice donor located downstream of the NPT gene in pRET, and the splice acceptor located upstream of exon 12 of the hamster PAPSS1 gene is shown. Since the hamster PAPSS1 gene has not been previously characterized by DNA sequencing, this sequence at the fusion junction is shown aligned with that of the corresponding mouse PAPSS1 exon 11-exon 12 junction nucleotide sequence. The amino acid sequence encoded by this region of mouse PAPSS1 is also shown. The nucleotide differences between the hamster and mouse sequences occur at codon wobble positions (indicated with lowercase letters) that do not alter the amino acid sequence. (B) IM2 cells were engineered to express human PAPSS1 or PAPSS2, or a control cDNA, as described under Materials and Methods. Lysates prepared from these cells were assayed for PAPSS activity by adding ATP and ^[35]^S labeled sulfate, and the samples were then subjected to thin layer chromatography to separate the substrate from the reaction products (APS and PAPS). The experiment shown is representative of three independent experiments. (C) The amounts of PAPS synthesized in the samples shown in panel C were quantitated as described under Materials and Methods and are shown relative to the amount produced by the wild-type CHO-K1 cell extract (defined as 100%). The data shown are the average of three independent experiments. Error bars indicate the standard deviation of the data.

To identify which cellular gene was disrupted by the mutagenic pRET vector, total RNA was isolated from IM2 cells and reverse transcription PCR amplification was performed using primers anchored on the virally encoded NPT gene and the poly (A) tail. DNA sequence analysis of the PCR amplification products and a comparison with the sequenced mouse genome revealed that the pRET provirus had integrated upstream of exon 12 of the 5′ phospho-adenosine, 3′phosphosulfate synthase 1 gene (PAPSS1) (Fig. 3A). The full sequence of hamster PAPSS1 gene, and its corresponding mRNA product, have not yet been reported. However, comparison with the cognate mouse gene indicates that, in IM2 cells, the pRET-encoded NPT open reading frame is fused by mRNA splicing to the third base of the codon encoding amino acid residue 579 of PAPSS1 (Fig. 3A).

PAPSS1 and the highly related PAPSS2 enzyme catalyze the formation of the high energy sulfate donor 3′ phospho-adenosine, 5′phosphosulfate (PAPS) [42],[47],[48] used for all sulfonation reactions in the cell. Consistent with the prediction that IM2 cells have less PAPS available for sulfonation reactions, IM2 cells incorporated 17% less ^[35]^SO_4_ into macromolecules than CHO-K1 cells in bulk labeling experiments (Figure S2). However, the readout of these experiments is several steps downstream of PAPS synthase and represents the summation of multiple enzyme/substrate interactions. To directly determine if IM2 cells were deficient in PAPS synthase activity, an in vitro PAPS assay was performed. ATP and ^[35]^SO_4_ were mixed with cell lysates prepared from CHO-K1 cells, IM2 cells, or IM2 cells engineered to express human cDNA clones of either PAPSS1 or PAPSS2. The reaction products were separated on PEI cellulose TLC plates in 0.9 M LiCl. (Fig. 3B) Inorganic sulfate exhibits the greatest mobility, followed by the reaction intermediate adenosine phosphosulfate (APS), with PAPS being retained closest to the origin [47]. Mobility positions were confirmed with commercial PAPS preparations (data not shown). TLC plates were exposed to phosphoimager plates and the levels of PAPS synthesized were measured. These studies demonstrated that IM2 cells have five-fold lower levels of PAPSS activity per µg of protein than do the parental CHO-K1 cells (Fig. 3B and 3C). PAPS synthase activity in IM2 cells was significantly increased by stable expression of either human PAPSS1 or PAPSS2 cDNA clones (Fig. 3B and 3C) although not to full WT levels. These data indicate that the pRET vector disrupted the function of the PAPSS1 gene in IM2 cells.

### The cellular sulfonation pathway is required for MLV infection

**Figure 4:**
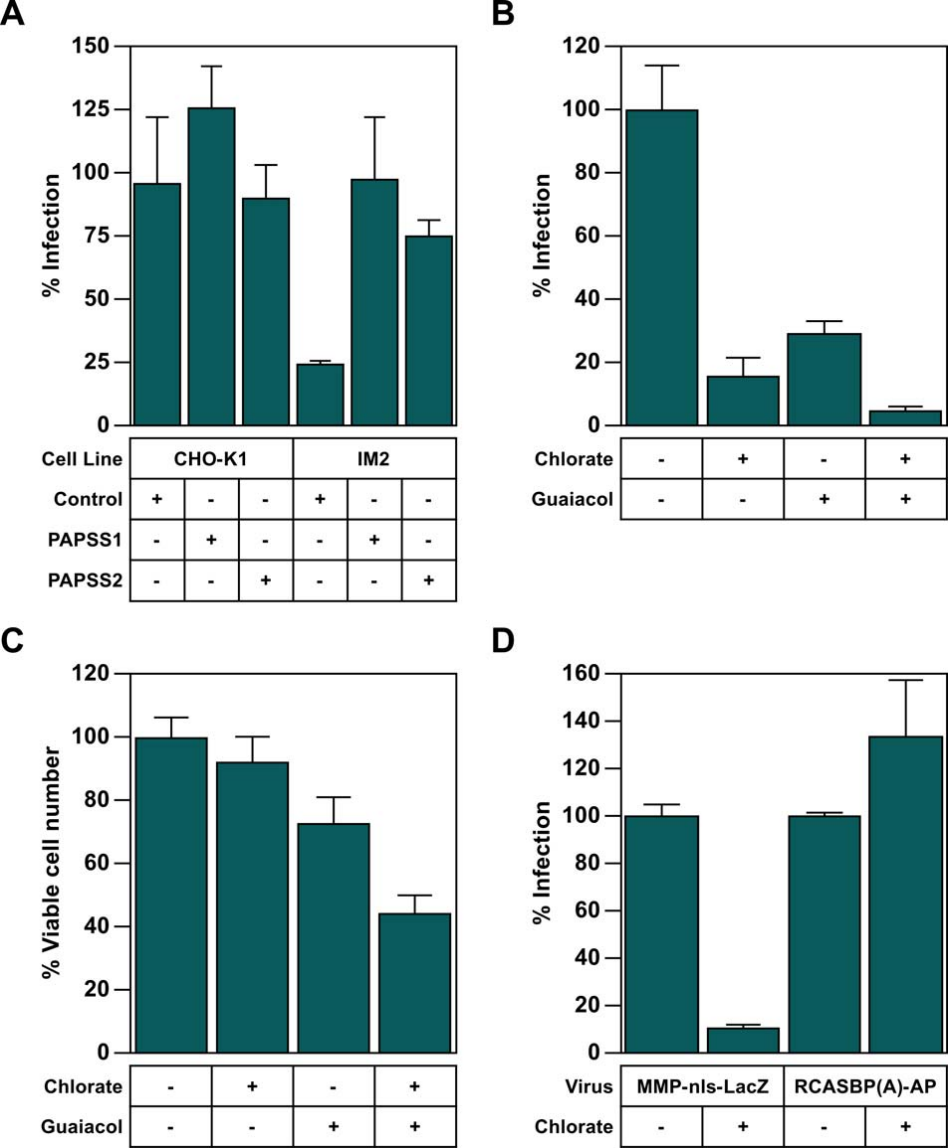
PAPSS activity is required for efficient MLV infection. (A) CHO-K1and IM2 cells engeneered to express either a control cDNA, human PAPSS1 or human PAPSS2 were challenged with serial dilutions of the VSV-G pseudotyped MLV vector (MMP-nls-lacZ[VSV-G]), encoding β-galactosidase. The cells were then stained 48 hpi with X-gal, the number of blue cells were counted, and the data reported as the percentage of LacZ transducing units (LTU) obtained from WT CHO-K1 infections (5×10^5^ LTU). The data shown are the average mean values obtained with triplicate samples. (B and C) CHO-K1 cells were challenged with a VSV-G pseudotyped MLV vector MMP-nls-lacZ[VSV-G]) in the presence of either 100 mM chlorate, 5 mM Guaiacol, or both 100 mM chlorate and 5 mM Guaiacol. The cells were subsequently assayed for either β-galactosidase activity using a chemiluminescent assay (B), or for viable cell number using a chemiluminescent assay (C). The data in panels B and C are the average mean values obtained in an experiment performed with quadruplicate samples and are reported as a percentage of that seen with untreated cells. (D) Chicken DF-1 cells were challenged with either the MLV vector pMMp-nls-LacZ[VSV-G], or the ASLV-A vector RCASBP(A)-AP in the presence of 100 mM chlorate and assayed 48 hpi with chemiluminescent assays for reporter enzyme activities or viable cell number. The ratios of [enzyme activities∶relative viable cell number] were calculated for each sample and compared with untreated controls (defined as 100% infection). The data shown are the average mean values obtained in an experiment performed with quadruplicate samples. The results shown in panels A–D are representative of three independent experiments and error bars indicate the standard deviation of the data.

To investigate whether the deficiency in PAPS synthase activity in IM2 cells was responsible for the block to MLV infection, CHO-K1 and IM2 cells engineered to express either human PAPSS1 or PAPSS2 were challenged with the VSV-G pseudotyped MLV vector encoding β-galactosidase and infected cells were enumerated by X-gal staining. Expression of either PAPSS enzyme complemented the MLV infection defect of the IM2 cell line (Fig. 4A). By contrast, a control cDNA, containing an ORF unrelated to sulfonation, did not rescue virus infectivity in these cells (Fig. 4A). These data confirm that the deficiency in PAPS synthase activity is responsible for the virus infection-resistant phenotype of IM2 cells.

To further investigate a role for the sulfonation pathway, CHO-K1 cells were treated with either chlorate, a substrate analog of sulfate and a competitive inhibitor of PAPS synthases [48]–[51], or with the sulfotransferase inhibitor guaiacol [50],[51], prior to challenge with the MLV vector. As compared to untreated cells, chlorate-treated, guiacol-treated, and chlorate/guaiacol dual-treated cells gave rise to approximately 6.7-fold, 3.4-fold, and 23-fold less blue cells, respectively (Fig. 4B). Only the dual inhibitor treatment led to a significant (2.3-fold) reduction in viable cell number (Fig. 4C), which was still considerably less than the effect on infection. Similarly, chicken DF1 cells treated with chlorate were approximately 9.3-fold less susceptible to infection by this viral vector as judged by reporter gene expression (Fig. 4D). However, this treatment did not influence infection of these avian cells by an ASLV-A vector. Treatment of IM2 cells with chlorate reduced MLV infection an additional 2-fold (Figure S3), which is consistent with the observation that these cells contain some residual PAPS synthase activity (Fig. 3B and 3C). These data further show a role for the cellular sulfonation pathway in infection by MLV, but not ASLV, vectors and indicate that the mechanism(s) responsible are shared between different host cell species.

### The sulfonation pathway does not influence viral reverse transcription, or the level of proviral DNA

**Figure 5:**
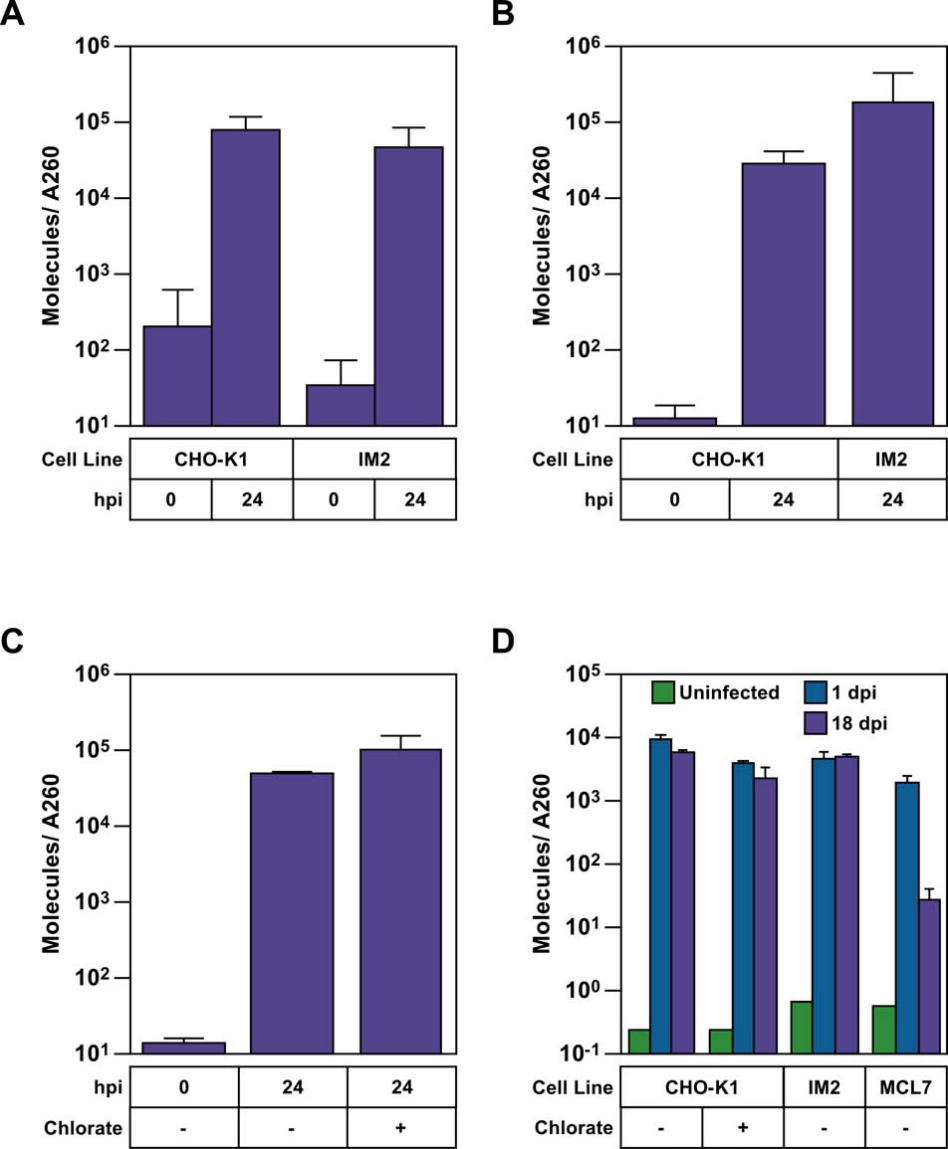
The sulfonation pathway does not influence reverse transcription or the level of integrated virus DNA. (A and B) CHO-K1 cell lines were challenged with the VSV-G pseudotyped MLV vector LEGFP. Total DNA (A) or DNA from isolated nuclei (B) were harvested at 0 or 24 hpi, DNA concentration was quantitated by A260, and a real-time PCR amplification analysis was performed to measure the levels of viral DNA that were synthesized. (C) Untreated CHO-K1 cells or CHO-K1 cells that had been pretreated for 16 hrs with 100 mM chlorate and maintained in medium containing this concentration of chlorate were challenged with the MLV vector and subsequently analyzed for reverse transcription products as described in panel A. (D) CHO-K1 cells that were either untreated or treated with 100 mM chlorate, IM2 cells, and MCL7 cells, were challenged with the same VSV-G pseudotyped MLV vector and total DNA was harvested at 1 or 18 days post infection for real time PCR quantitation of viral DNA products. The chemically-mutagenized MCL7 cell line displays a strong block to MLV DNA integration [39]. Chlorate treated CHO-K1 cells were passaged in medium containing 100 mM chlorate for the duration of the experiment. The data shown in panels A–D are the average mean values obtained in independent experiments performed with triplicate samples and each is representative of three independent experiments. Error bars indicate the standard deviation of the data.

Real time PCR amplification was used to monitor the effect of the sulfonation pathway on the levels of reverse transcription products and integrated viral DNA. Cells were infected with an MLV vector (pLEGFP) and either total DNA or nuclear DNA was subsequently harvested. Since these cells potentially contain both the mutagenic pRET vector, and the pCMMP derived vector utilized in the screen, the primer/probe set was chosen to amplify the plus strand strong stop replication intermediate [52],[53] and annealed specifically to the unique 3 (U3) and unique 5 (U5) long terminal repeat (LTR) region of only the pLEGFP MLV vector (data not shown). This primer probe set exhibits an excellent dose response over 6 orders of magnitude (Figure S4A) and a very low background, such that the signal from infected cells at 24 hpi is 400-fold higher than from cells where the virus is bound but not internalized (0 hpi, Fig. 5A) The difference between infected and uninfected cells is even greater (Fig. 5D and Figure S4B). The levels of total and nuclear reverse transcription products were found to be the same in IM2 cells as in CHO-K1 cells (Fig. 5A and 5B). Furthermore, treatment of CHO-K1 cells with chlorate had no effect on the accumulation of viral reverse transcription products, confirming that the sulfonation pathway does not influence viral DNA synthesis (Fig. 5C). Importantly, this is not due to saturation of the assay as dilution of input genomic DNA showed a proportionate decrease in both CHO-K1 and IM2 samples, even when a ten-fold higher multiplicity of infection was used (Figure S4B). To investigate the possible role of this pathway in viral DNA integration, IM2 cells were infected with the same MLV vector, passaged for 18 days to allow loss of episomal forms of viral DNA [54],[55], and the levels of total viral DNA were then measured. IM2 cells and chlorate treated CHO-K1 cells contained nearly the same amounts of integrated viral DNA as untreated CHO-K1 cells (1.1 and 2.6-fold less, respectively) (Fig. 5D), which is insufficient to explain the approximately 10-fold decrease in infectivity (Fig. 2A and 4B). By comparison at 18 days post-infection, nearly 400-fold lower levels of viral vector DNA were detected in MCL7 cells, a chemically mutagenized CHO-K1 cell line that exhibits a strong block to MLV DNA integration [39] (Fig. 5D). These data demonstrate that the cellular sulfonation pathway does not influence either the levels of viral DNA that are synthesized in the target cell or that become integrated into the host cell genome.

### The sulfonation pathway influences gene expression from the MLV LTR

**Figure 6:**
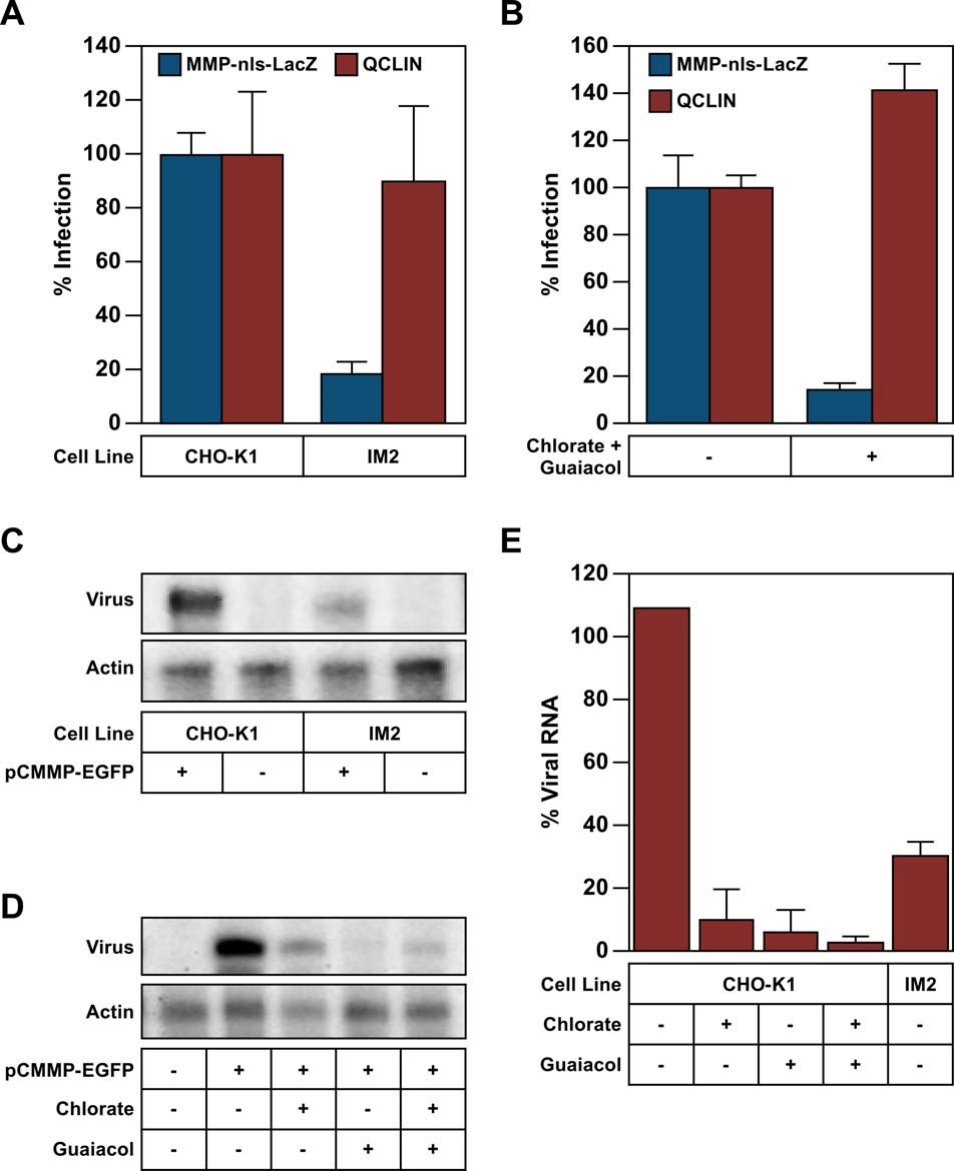
The sulfonation pathway influences transcription specifically from the MLV LTR. (A) CHO-K1 or IM2 cells were challenged with the VSV-G pseudotyped MLV vectors MMP-nls-lacZ, which directs MLV LTR-driven lacZ gene expression, or with pQCLIN, a self-inactivating MLV vector with defective LTRs and an internal CMV promoter which drives lacZ expression. The cells were subsequently assayed for β-galactosidase activity and viable cell number and the data reported as in Fig. 2B with the ratio of [β-galactosidase activity: relative viable cell number] observed with CHO-K1 cells defined as 100% infection. (B) CHO-K1cells were challenged with the same virus vectors used in panel A but in the presence of 100 mM chlorate and 5 mM Guaiacol. The cells were subsequently assayed for β-galactosidase activity as in panel A. The data shown in panels A and B are the average mean values obtained in an experiment performed with quadruplicate samples and each is representative of three independent experiments. (C) Total RNA was isolated from CHO-K1 or IM2 cells that had been infected with the VSV-G pseudotyped MLV vector pCMMP- EGFP. RNase protection assays were performed using a probe that recognizes either the provirus-derived transcript or the hamster β-actin gene. Protected fragments were separated, subjected to gel electrophoresis as described under Materials and Methods, and exposed to a phosphoimager plate. (D) CHO-K1 cells were challenged with virus as in panel C in the presence of either 100 mM chlorate, 5 mM guaiacol, or with both inhibitors. The inhibitors were present throughout the infection. (E) The mean average data of at least three independent RNase protection experiments, conducted as in panels C and D, were quantitated by phosphorimaging using the image quant software volume method. The relative levels of viral transcripts were normalized to the corresponding β-actin levels and are reported as a percentage of those seen with untreated CHO-K1 cells (defined as 100%). Error bars in panels A, B and E, indicate the standard deviation of the data.

Since the sulfonation pathway did not influence the level of integrated viral DNA, we next determined if it impacts subsequent provirus gene expression. In these studies, the level of MLV LTR-driven transcription from the MMP-nls-LacZ vector was compared to that from the internal CMV promoter contained in QCLIN, a commercially available, self-inactivating (SIN) MLV vector with promoter defective LTRs [56]. The levels of β-galalactosidase from the QCLIN vector were the same in infected IM2 and CHO-K1 cells (Fig. 6A), a result that supports our observation that the sulfonation pathway does not influence the overall level of viral DNA integration. By striking contrast, MLV LTR-driven reporter gene expression following infection was reduced 5.6-fold in IM2 cells as compared with CHO-K1 cells (Fig. 6A). Consistently, a combination of chlorate and guaiacol treatment reduced β-galalactosidase levels produced from the MMP-nls-lacZ vector by 7.3-fold, following infection of CHO-K1 cells, but this treatment did not influence gene expression from the SIN vector (Fig. 6B). These data suggest that the target of action for the cellular sulfonation pathway is contained within the MLV LTR.

To directly examine the influence of the sulfonation pathway upon MLV LTR-driven mRNA transcription, total RNA was isolated from CHO-K1 and IM2 cells that were infected with a VSV G-pseudotyped MLV vector encoding EGFP. RNase protection assays were performed with a probe that hybridizes to the primary viral mRNA transcript (hybridizing to the R-U5 region). IM2 cells accumulated 3.5-fold less primary transcript than CHO-K1 cells (Fig. 6C & E). Similarly, the levels of viral-derived transcript were reduced in CHO-K1 cells treated with inhibitors of the cellular sulfonation pathway (Chlorate 11- fold, guaiacol 17- fold, and chlorate and guaiacol 40- fold (Fig. 6D & E). All values were normalized to hamster β-actin levels, which varied less than 2-fold in all cases (Fig. 6C and 6D). These data indicate that the sulfonation pathway influences a step that impacts the transcriptional competency of the provirus.

### The sulfonation pathway acts at a step during provirus establishment

**Figure 7:**
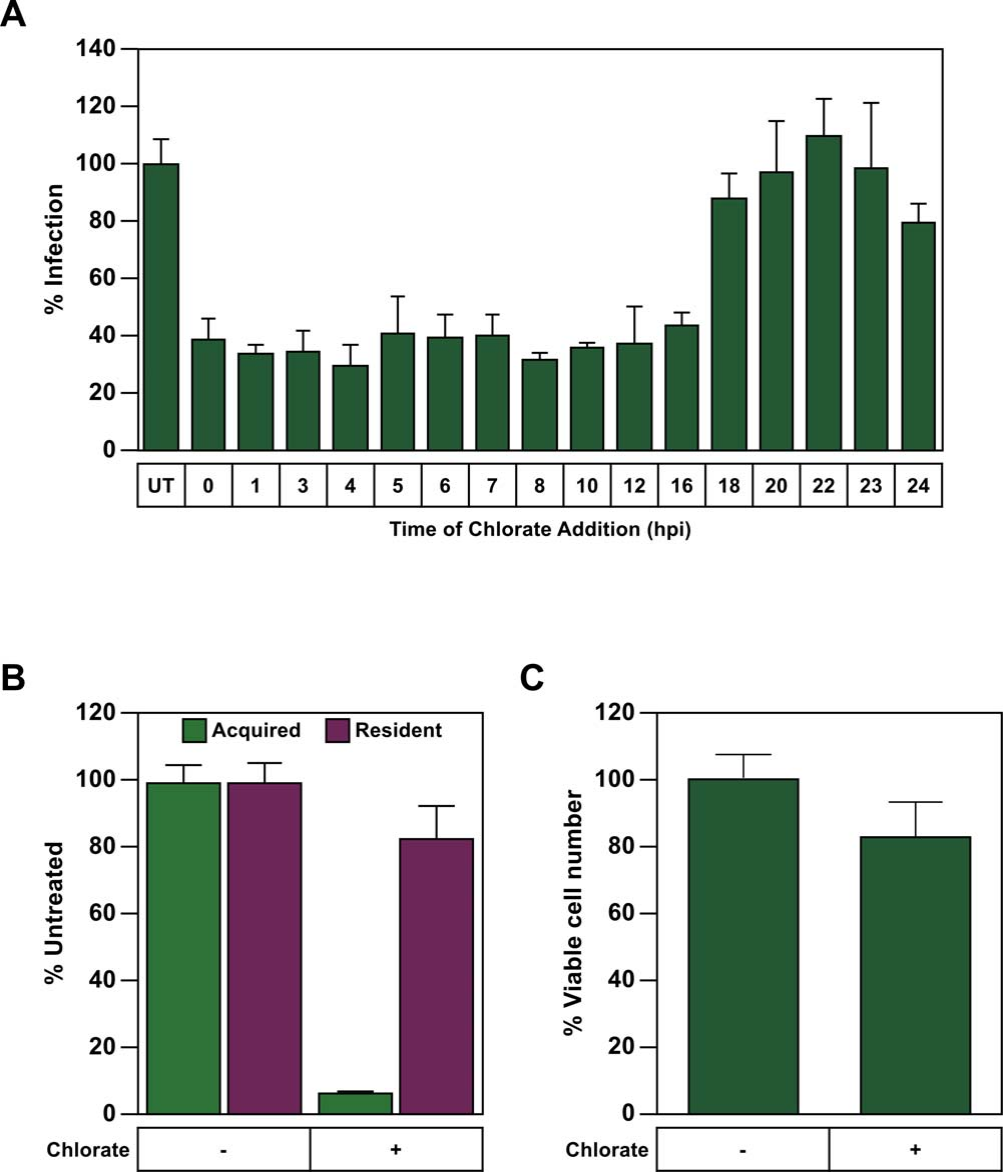
Chlorate treatment during virus infection reduces reporter gene expression from newly acquired, but not resident proviruses. (A) CHO-K1 cells (1×10^5^) were challenged with 5×10^5^ IU of MMP-nls-lacZ[VSV-G] at 4°C for 2 hrs and then warmed to 37°C to initiate infection at t = 0 mins. Chlorate was added to 100 mM final concentration at the indicated hours post infection (hpi). At 48 hpi, the cells were assayed for β-galactosidase activity and for relative viable cell numbers and the level of infection was calculated as in Fig. 2B. The data shown are the average mean values obtained in an experiment performed with triplicate samples. (B) Cells harboring a resident MLV provirus, pCMMP-SEAP, encoding secreted alkaline phosphatase, were challenged with a second MLV vector, MMP-nls-lacZ[VSV-G] encoding β-galactosidase, in the presence of 100 mM chlorate. Chemiluminescent assays were then used to measure reporter enzyme activity levels (panel B), as well as relative viable cell numbers (panel C), and the values obtained with untreated cells was defined as 100% in each case. The data shown in panels C and D are the average mean values obtained in an experiment performed with quadruplicate samples. The data in panels A–C are representative of three independent experiments and error bars indicate the standard deviation of the data.

To determine the time point during infection when the cellular sulfonation pathway is involved, CHO-K1 cells were incubated with the VSV-G pseudotyped MLV vector encoding β-galactosidase at 4°C, and infection was then initiated by a temperature shift to 37°C. Chlorate was then added at various times post-infection and the effect of this treatment on the establishment of viral vector in these cells was then measured by quantitating β-galalactosidase expression. Chlorate addition up to 16 hpi led to a reduction in subsequent viral reporter gene expression (Fig. 7A). However, addition of the inhibitor at time points 18 hpi, or later, had no effect (Fig. 7A). This timing coincides with maximal levels of viral DNA integration [57], suggesting that the cellular sulfonation pathway might influence a step during or shortly after provirus establishment.

To explore this possibility further we compared the effect of chorate treatment on proviral gene expression from resident, versus newly acquired, proviruses. A CHO-K1 cell line was established that contains a resident MLV vector encoding secreted alkaline phosphatase (Fig. 7B). These cells were then challenged with the MLV vector encoding β-galactosidase in the presence of chlorate to generate newly acquired MLV proviruses under conditions where the sulfonation pathway was inhibited. These experiments showed that the chlorate treatment affected gene expression from the newly acquired, but not the resident proviruses (Fig. 7B). In an independent experiment, chlorate treatment was shown not to influence β-galalactosidase expression from a resident MLV vector (data not shown), confirming that the effect seen was not reporter gene-specific. Taken together with the timing of the sulfonation requirement during infection (Fig. 7A), these results strongly imply that this cellular pathway influences MLV replication at a step during provirus establishment, one that impacts subsequent viral gene expression.

### The sulfonation pathway affects HIV LTR-driven transcription

**Figure 8:**
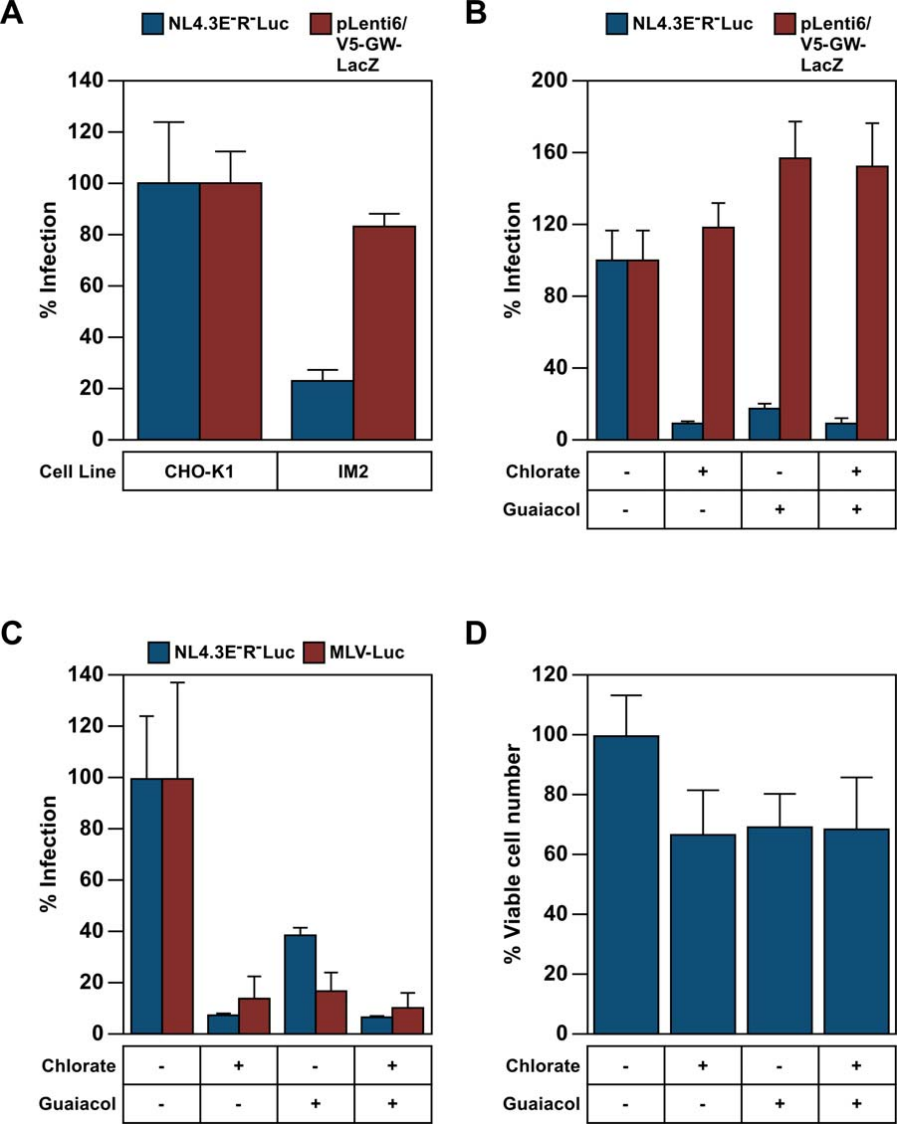
PAPSS activity affects transcription from the HIV-1 LTR. (A) CHO-K1 or IM2 cells were challenged with either one of two VSV-G pseudotyped HIV-1 vectors, NL43E-R-Luc, that directs luciferase gene transcription from the viral LTR, or with pLenti6/V5-GW/LacZ, a self inactivating HIV-1 vector from which β-galactosidase expression is driven by an internal CMV promoter. Chemiluminescent assays were used to measure reporter enzyme activities and viable cell numbers. The data shown was calculated as in panel Fig. 2B and the value obtained with CHO-K1 cells was defined as 100% infection. (B) CHO-K1 cells were challenged with the viruses described in panel A, in the presence of either 100 mM chlorate, 5 mM Guaiacol or both inhibitors and subsequently assayed as in panel A. (C) Jurkat cells were spin inoculated with the VSV-G pseudotyped vectors NL43E-R-Luc[VSV-G] or MLV-LUC (an MLV vector that encodes luciferase) in the presence of 120 mM Chlorate, 5 mM guaiacol or both inhibitors. Chemiluminescent assays were used to monitor virus infection as in panel A. The viable cell number observed in the experiment shown in panel C is reported in panel D. For panels B–D the data are reported as the percentage of untreated controls. For panels A–D, the data is the average mean values obtained in an experiment with quadruplicate samples and are representative of three independent experiments. Error bars indicate the standard deviation of the data.

The previous experiments showed that the sulfonation pathway affects LTR-driven gene expression from newly acquired MLV, but not ASLV, proviruses. To test the influence of this pathway on HIV-1 LTR-driven gene expression, CHO-K1 and IM2 cells were challenged with either of two VSV-G pseudotyped HIV-1 vectors, one with luciferase expressed from the viral LTR and the other a SIN vector with β-galactosidase expressed from an internal CMV promoter. Reporter gene expression from the HIV-LTR was reduced 5-fold in IM2 versus CHO-K1 cells whereas that from the internal CMV promoter was the same in both cell types (Fig. 8A). Consistently, treatment of CHO-K1 cells with chlorate, guaiacol, or with both inhibitors resulted in 10-, 8-, and 12-fold reductions in HIV LTR-driven reporter gene expression, respectively. By contrast, internal CMV promoter-driven reporter gene expression was unaltered or was slightly enhanced by these treatments (Fig. 8B). Similar results were observed using human Jurkat T cells infected with VSV-G pseudotyped HIV or MLV viral vectors that express luciferase from the viral LTRs (Fig. 8C). Therefore as for MLV, HIV LTR-driven gene expression is also regulated by the cellular sulfonation pathway.

## Discussion

Here we have presented multiple lines of evidence that the host cell sulfonation pathway influences retroviral infection by affecting a step during provirus establishment, one that modulates gene expression from the viral LTR promoter. First, insertional mutagenesis and genetic complementation studies identified PAPSS1 as a cellular gene that is important for MLV infection. Second, a similar defect was seen with cells treated with the PAPS synthetase inhibitor, chlorate, or with the sulfotransferase inhibitor, guaiacol. Third, inhibition of the sulfonation pathway had no impact on the levels of integrated MLV DNA but influenced downstream MLV LTR-driven gene expression from newly formed proviruses. Fourth, MLV was sensitive to inhibitors of the sulfonation pathway at time points up to that associated with maximal levels of viral DNA integration [57]. Finally, the observations made with MLV held true for HIV-1, the causative agent of AIDS, since the sulfonation pathway also influenced LTR-driven transcription from that virus. These data suggest that sulfonation may play an important role in the regulation of nuclear gene expression. Consistent with this, PAPSS1 localizes to the nucleus, which implies there is a requirement for high levels of PAPS, and by extension sulfonation, in the nucleus [58]. Thus, these studies have uncovered a heretofore unknown regulatory step of retroviral replication, one that is potentially important for HIV/AIDS therapy.

The data in this report are consistent with either one of two models. In the first model, the sulfonation pathway might influence viral DNA integration site specificity so that when this pathway is impaired, the virus is targeted to regions where the provirus is less transcriptionally competent. This model is consistent with the observation that viruses sensitive to the sulfonation pathway, HIV and MLV, both share a strong preference for integration into genes, although MLV shows a much stronger preference for integration near the viral promoter regions [59]–[62]. By contrast, ASLV, which is not influenced by this pathway, shows little or no preference for integration into genes [60],[63].

In the second model, the sulfonation pathway might have no impact upon integration site specificity but, during integration or shortly thereafter, the sulfonation pathway might influence the nature of epigenetic modifications introduced onto the viral DNA. These modifications could, in turn, regulate the transcriptional competency of the provirus. Sulfonation induced changes in DNA methylation, histone acetylation, methylation or positioning are all potential processes which could affect the transcriptional activity of the provirus [64],[65]. Indeed the importance of epigenetic modifications in HIV transcription is apparent in a recent large scale analysis of HIV integration sites which revealed a positive correlation between integration and epigenetic modifications favoring transcription and a negative correlation with modifications that silence transcription [66]. We are currently performing experiments aimed at distinguishing between these two models.

The host cell sulfonation pathway involves a set of golgi and cytoplasmic sulfotransferases (SULTs) that transfer the sulfonate from PAPS to target substrates. In humans there are thirteen distinct cytosolic SULTs, arranged into three different families, and these enzymes are involved in the metabolism of steroids, bile acids, neurotransmitters, and xenobiotics [67]. Golgi sulfotransferases are involved in sulfonating carbohydrates, generating the glycosaminoglycans (GAGs), heparan sulfate, chondroitin/dermatan sulfate, and keratan sulfate [68], as well as glycolipids [29]. Two golgi tyrosylprotein sulfotransfrerases (TPST-1 and TPST-2) are responsible for sulfonation of tyrosine residues on proteins and peptides. Tyrosyl sulfonation can have important regulatory effects on cell surface proteins including an influence on protein-protein interactions [69], as exemplified by the requirement for sulfonation of tyrosine residues at the amino-terminus of the CCR5 chemokine receptor for high affinity interaction with both its natural ligands, MIP-1α and MIP-1β, as well as with HIV-1 gp120 [70]. This entry effect seen previously is distinct from our observation that sulfonation also affects a post entry step coinciding with provirus establishment. Since inhibition of sulfonation can block HIV at multiple stages of the viral lifecycle, the cellular sulfonation pathway is an intriguing target for the development of novel antivirals.

Future work will be aimed at identifying the specific components of the sulfonation pathway that are critical for modulating MLV and HIV-1 infection. We expect that this information will help to uncover precisely how the sulfonation pathway regulates retroviral infection at a step coincident with provirus establishment and that influences the subsequent transcriptional competency of the provirus.

## Supporting Information

Figure S1 Schematic diagrams of the proviral forms of MLV vectors used in this report. A. Following integration of the pRET vector in a reverse orientation within an intron of a cellular gene, mRNA splicing gives rise to an IRES-containing transcript that encodes GFP. An internal promoter drives expression of the neomycin phosphotransferase gene (NPT) which confers G418 resistance only when a downstream mRNA instability motif is removed by splicing to a downstream cellular exon. A downstream poly (A) signal derived from the cellular gene is captured to stabilize the RNA. B. The MLV pCMMP based vectors have the gag/pol and env genes replaced with various reporter genes, including CD4, HcRed, LacZ, and luciferase. Reporter gene expression is driven from the viral LTR. The MLV vector pLEGFP has a similar structure but has both WT LTRs and an internal CMV promoter driving GFP expression. C. The self-inactivating MLV vector pQLIN has the U3 elements of the viral LTRs deleted and the gag/pol and env genes replaced with the CMV promoter driving expression of the LacZ gene.
(0.49 MB TIF)

Figure S2 Total sulfonation of macromolecules is reduced in IM2 cells. CHO-K1 and IM2 cells were incubated in sulfate free media supplemented with 200 μCi/ml ^[35]^SO_4_ for 48 hours. Cells were then harvested in 50 mM Tris-pH 7.0, 2% SDS. The samples were then precipitated in 25% TCA. The precipitates were washed 3× with 5% TCA, once with 95% ethanol and air dried. Samples were suspended in scintillation fluid and counted for 1 minute, and incorporation of label was normalized to CHO-K1 values. Values shown are the average of quadruplicate sample readings from two independent labeling experiments. Error bars indicate the standard deviation of the data.
(0.11 MB TIF)

Figure S3 Effect of chlorate on MLV vector infection of IM2 cells. IM2 cells were challenged with the MLV vector pMMp-nls-LacZ[VSV-G] in the presence of 120 mM chlorate and assayed 48 hpi with chemiluminescent assays for reporter enzyme activities or viable cell number. The ratios of [enzyme activities:relative viable cell number] were calculated for each sample and compared with untreated controls (defined as 100% infection). The data shown are the average mean values obtained in an experiment performed with quadruplicate samples. The results are representative of three independent experiments and error bars indicate the standard deviation of the data.
(0.11 MB TIF)

Figure S4 Standard curve of real-time quantitative PCR analysis. Serial dilutions of pLEGFP-C1 plasmid were amplified as described in materials and methods and the threshold cycle value for each dilution was plotted against the number of input molecules of DNA. Non-linear regression analysis was performed on the data and the r^2^ value was used to determine the fit of the data. This data was used to generate the standard curve for Figure 5D. (B) To determine if the QPCR assay was linear under the conditions of our analysis, 1×10^6^ cells were infected at an m.o.i. of 10 (10 times higher than the amounts used in our standard assay conditions in Fig. 5), total DNA was isolated and then real time PCR analysis was performed as described in materials and methods using the indicated dilutions of input viral DNA (used as a surrogate marker of the number of virions added). The data shown are the average mean values obtained in independent experiments performed with triplicate samples and each is representative of three independent experiments. Error bars indicate the standard deviation of the data.
(0.39 MB TIF)
