# Supplementary figures and images for: The Host Cell Sulfonation Pathway Contributes to Retroviral Infection at a Step Coincident with Provirus Establishment
Source: PLoS Pathog. 2008 Nov 14;4(11):e1000207. doi: 10.1371/journal.ppat.1000207 (PMC2576444; doi:10.1371/journal.ppat.1000207)

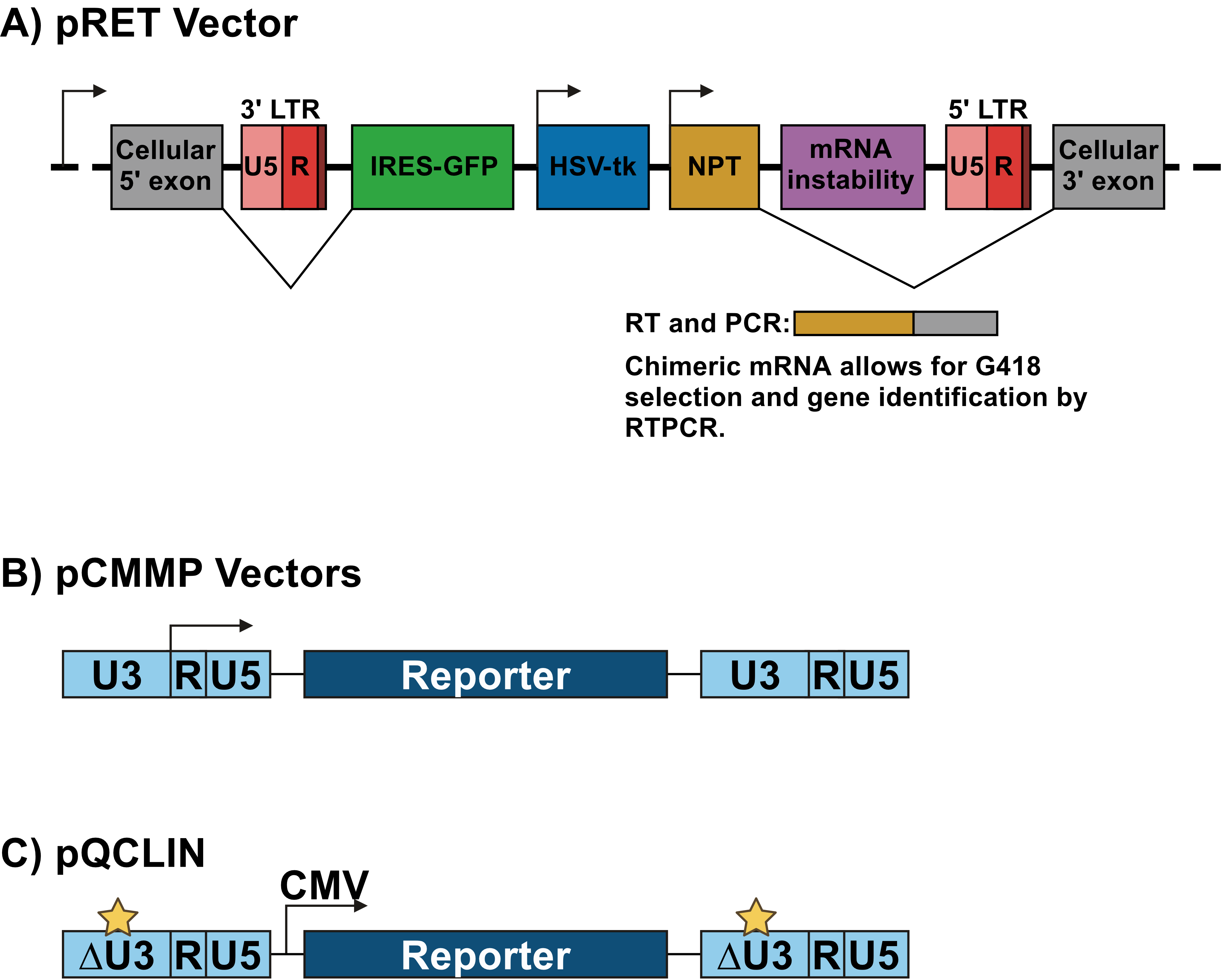

Supplement: Figure S1 — Schematic diagrams of the proviral forms of MLV vectors used in this report. A. Following integration of the pRET vector in a reverse orientation within an intron of a cellular gene, mRNA splicing gives rise to an IRES-containing transcript that encodes GFP. An internal promoter drives expression of the neomycin phosphotransferase gene (NPT) which confers G418 resistance only when a downstream mRNA instability motif is removed by splicing to a downstream cellular exon. A downstream poly (A) signal derived from the cellular gene is captured to stabilize the RNA. B. The MLV pCMMP based vectors have the gag/pol and env genes replaced with various reporter genes, including CD4, HcRed, LacZ, and luciferase. Reporter gene expression is driven from the viral LTR. The MLV vector pLEGFP has a similar structure but has both WT LTRs and an internal CMV promoter driving GFP expression. C. The self-inactivating MLV vector pQLIN has the U3 elements of the viral LTRs deleted and the gag/pol and env genes replaced with the CMV promoter driving expression of the LacZ gene. (0.49 MB TIF) [file ppat.1000207.s001.tif]

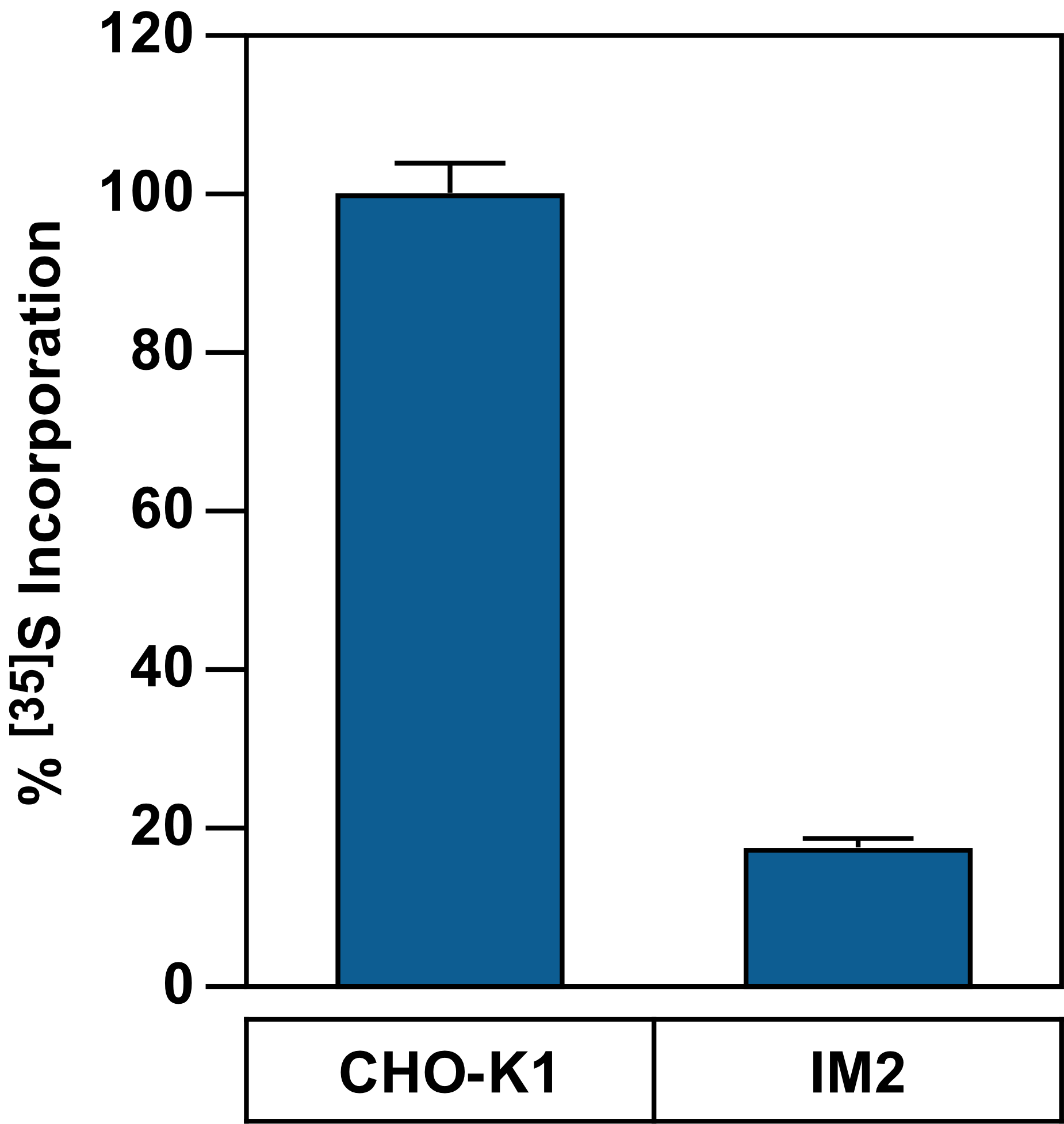

Supplement: Figure S2 — Total sulfonation of macromolecules is reduced in IM2 cells. CHO-K1 and IM2 cells were incubated in sulfate free media supplemented with 200 μCi/ml [35]SO4 for 48 hours. Cells were then harvested in 50 mM Tris-pH 7.0, 2% SDS. The samples were then precipitated in 25% TCA. The precipitates were washed 3× with 5% TCA, once with 95% ethanol and air dried. Samples were suspended in scintillation fluid and counted for 1 minute, and incorporation of label was normalized to CHO-K1 values. Values shown are the average of quadruplicate sample readings from two independent labeling experiments. Error bars indicate the standard deviation of the data. (0.11 MB TIF) [file ppat.1000207.s002.tif]

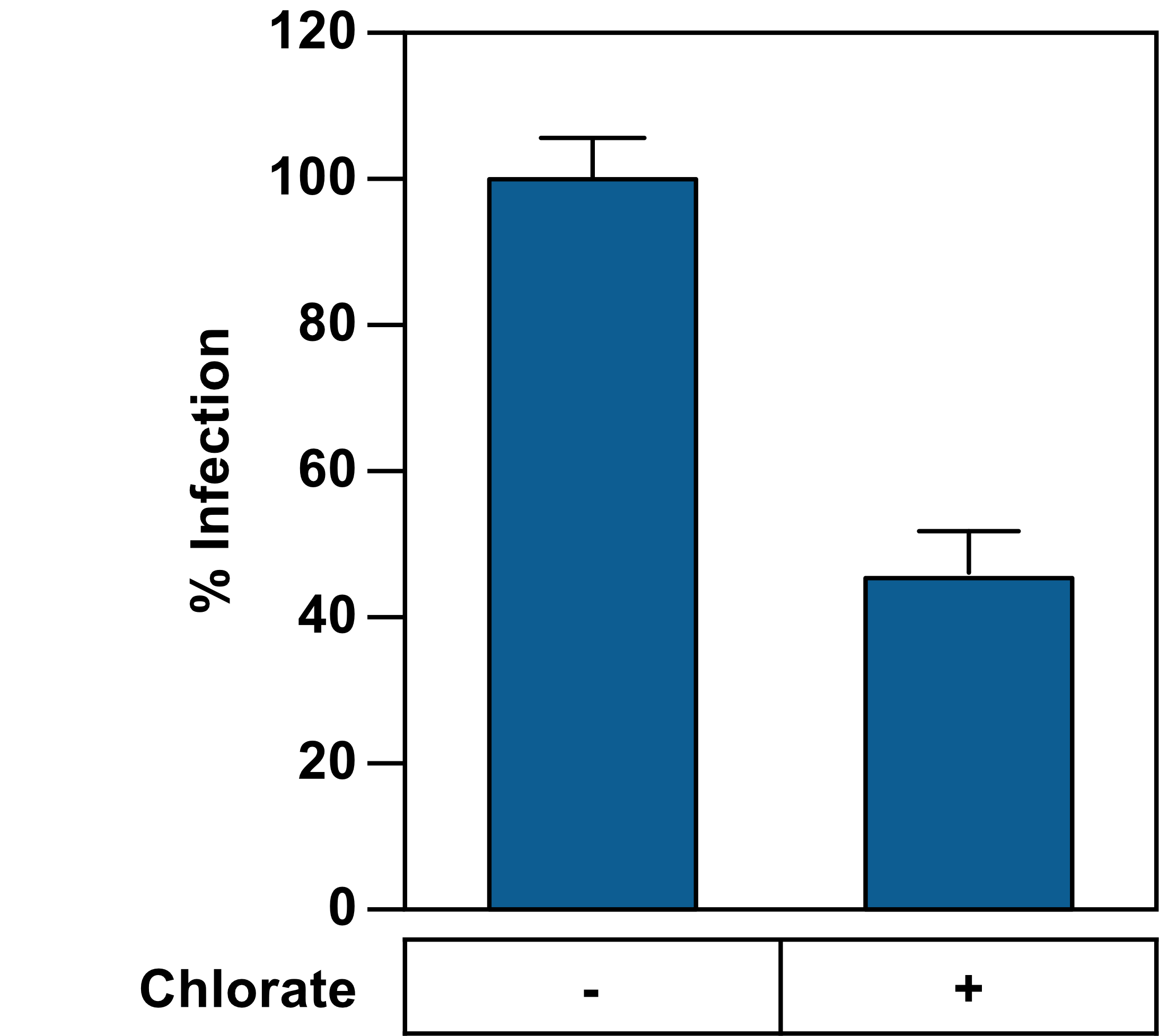

Supplement: Figure S3 — Effect of chlorate on MLV vector infection of IM2 cells. IM2 cells were challenged with the MLV vector pMMp-nls-LacZ[VSV-G] in the presence of 120 mM chlorate and assayed 48 hpi with chemiluminescent assays for reporter enzyme activities or viable cell number. The ratios of [enzyme activities:relative viable cell number] were calculated for each sample and compared with untreated controls (defined as 100% infection). The data shown are the average mean values obtained in an experiment performed with quadruplicate samples. The results are representative of three independent experiments and error bars indicate the standard deviation of the data. (0.11 MB TIF) [file ppat.1000207.s003.tif]

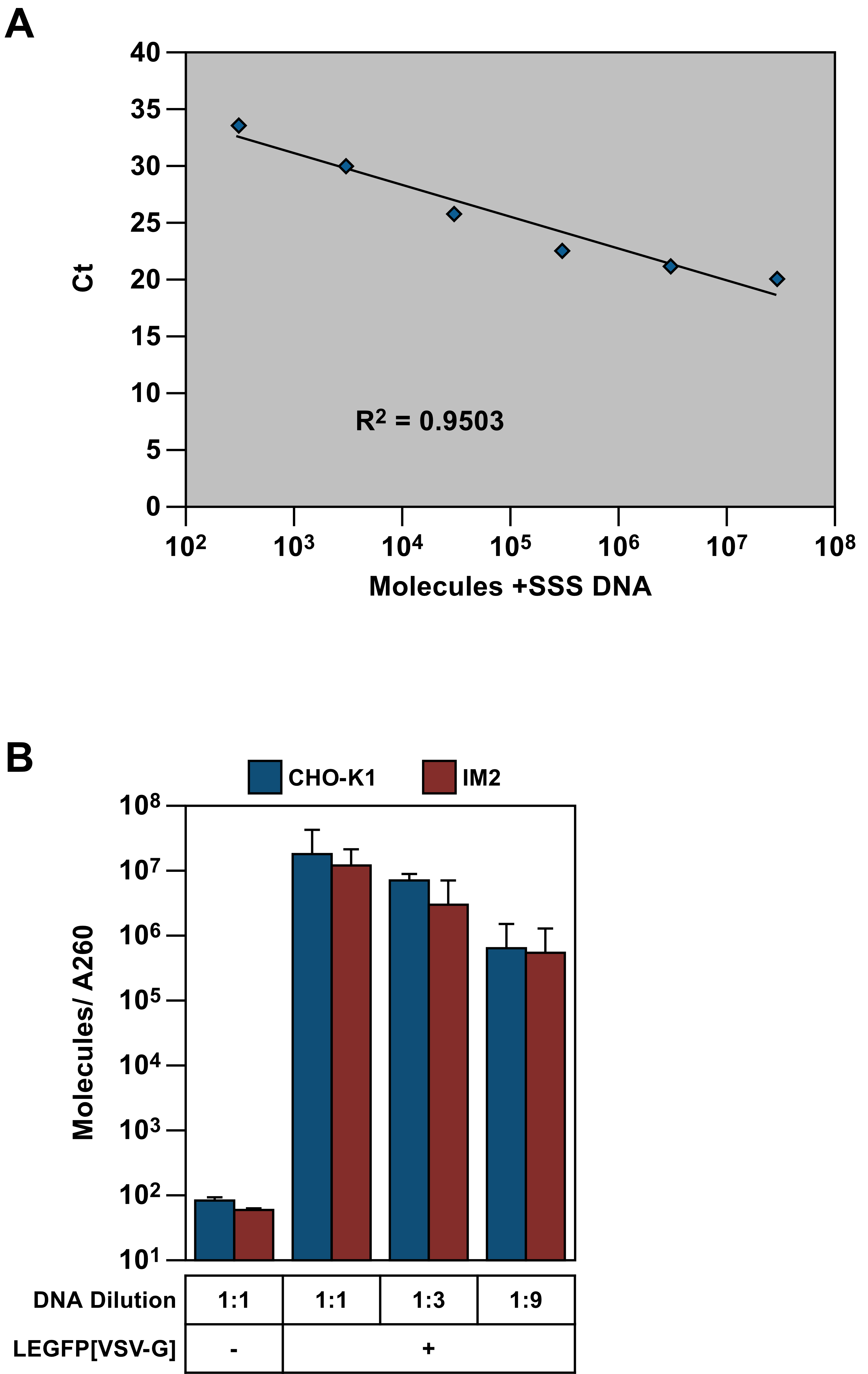

Supplement: Figure S4 — Standard curve of real-time quantitative PCR analysis. Serial dilutions of pLEGFP-C1 plasmid were amplified as described in materials and methods and the threshold cycle value for each dilution was plotted against the number of input molecules of DNA. Non-linear regression analysis was performed on the data and the r2 value was used to determine the fit of the data. This data was used to generate the standard curve for Figure 5D. (B) To determine if the QPCR assay was linear under the conditions of our analysis, 1×10^6^ cells were infected at an m.o.i. of 10 (10 times higher than the amounts used in our standard assay conditions in Fig. 5), total DNA was isolated and then real time PCR analysis was performed as described in materials and methods using the indicated dilutions of input viral DNA (used as a surrogate marker of the number of virions added). The data shown are the average mean values obtained in independent experiments performed with triplicate samples and each is representative of three independent experiments. Error bars indicate the standard deviation of the data. (0.39 MB TIF) [file ppat.1000207.s004.tif]
